# Industry relationships among authors of U.S. Clinical Practice Guidelines in cardiology from 2014 to 2020

**DOI:** 10.1016/j.heliyon.2023.e17309

**Published:** 2023-06-15

**Authors:** Subhanik Purkayastha, Ruina Zhang, Xiaohan Ying, Vinay Kini

**Affiliations:** a Weill Cornell Medicine, Department of Medicine, New York, NY, USA; b Weill Cornell Medicine, Division of Cardiology, New York, NY, USA

**Keywords:** Industry relationships, Clinical practice guideline authors

## Abstract

Clinical practice guidelines and Scientific statements are influential publications that define the standard of care for many diseases. However, little is known about industry payments and financial conflict-of-interest among authors of such publications in cardiology. We identified guidelines published between 2014 and 2020 by the American Heart Association (AHA) and the American College of Cardiology (ACC) in order to assess the payment status of CPG authors using the Open Payment Program (OPP) database.

## 1 Introduction

Clinical practice guidelines and Scientific statements, hereby referred to generally as guidelines, are influential publications that define the standard of care for many diseases. In recent years, there has been an increase in relationships with industry (RWI) in academia to conduct large, randomized trials of new medicines and technologies. While some level of RWI is necessary to test potential new advances in medical care, the National Academy of Medicine has published policies to regulate industry influence on the development of guidelines [1], since financial conflicts of interest (FCOI) between guideline authors and industry create the potential to introduce bias. Prior studies have shown a high prevalence and monetary value of industry payments to guideline authors in some fields, as well as failure to properly disclose some payments [[2], [3], [4]]. However, little is known about FCOI among guideline authors in cardiology, despite the significant burden of cardiovascular disease in the U.S. and the rapid pace of cardiovascular technological and pharmaceutical innovation. Accordingly, we examined industry payments received and FCOI disclosed by physician authors of cardiology guidelines.

## 2 Methods

We identified and reviewed both Clinical Practice Guidelines and Scientific Statements published between 2014 and 2020 by the American Heart Association (AHA) and the American College of Cardiology (ACC) by accessing the ACC and AHA guideline archives [5,6]. We extracted the names of cardiologists practicing in the United States who participated in the writing of guidelines as well as their disclosed FCOIs during this period. General industry payments between January 2014 and December 2020 were obtained from Open Payments Program (OPP), a publicly available database created by the Centers for Medicare and Medicaid Services (https://openpaymentsdata.cms.gov). We linked the OPP database with the National Plan and Provider Enumeration System (NPPES) database that contains physicians’ National Provider Identifier in order to associate guideline authors to their respective payments (https://npiregistry.cms.hhs.gov/).

First, we reported descriptive statistics of industry payments received by guideline authors from 2014 to 2020, including all payment years for each guideline. We included all payment years to understand industry relationships prior to guideline development as well as payments received following publication, since 1) guidelines can take years from conception to publication and 2) authors may gain status and expertise following publication which can result in future speaking or consulting fees. Next, we assessed payments received by guideline authors within 2 years of guideline publication, since this approach may evaluate potential bias in guidelines more directly. Since annual industry payment data was unavailable in OPP until 2014, we limited this analysis to guidelines published between 2016 and 2020. We calculated the proportion of all guideline authors who received payments, the total value of payments, and the number of authors who received payments at the following levels: <$100; $100-$999; $1000-$9999; $10,000-$99,999; $100,000-$999,999; and >$1,000,000. We also calculated the annual percent change in the proportion of guideline authors who received payments and the median sum of payments per guideline. Finally, we evaluated whether FCOI disclosures made by guideline authors at the time of guideline publication correlate with OPP database records.

## 3 Results

Among 578 physicians who co-authored 41 guidelines between 2014 and 2020, 169 (29%) received at least one industry payment. Payments to guideline authors totaled $16.8 million, or $29,107 per person (median, $11,232; IQR, $802-79,609). The proportion of authors with industry payments per guideline was 39% (IQR, 14%–61%). Of authors who received payments, 82 (14.2%) received less than $10,000, whereas 38 (6.6%) received more than $100,000. These 38 authors accounted for $14.8 million, or 87.8% of total payments.

Of the 430 physicians who co-authored 26 guidelines between 2016 and 2020, 102 (24%) authors received payments within 2 years of guideline publication. Payments made to guideline authors within 2 years of guideline publication totaled $4.3 million, or an average of $41,823 per person (median, $4050; IQR, $382-40,181). The median percentage of authors per guideline who received payments within 2 years of publication was 21% (IQR, 6%–42%). The number of authors receiving payments and the median value of payments per guideline are shown in Table 1. From 2016 to 2020, there were no significant changes in the proportion of authors who received payments (APC -11.3%; p = 0.60) or the median sum of payments (APC -60.5%; p = 0.05). Among the 102 authors who received payments within 2 years of publication of their respective guidelines, 56 (55%) disclosed FCOI at the time of guideline publication. Of the 46 authors who failed to disclose FCOI, 18 (39%) only received minor payments of $250 or less.

**Table 1:** General industry payments received by authors of cardiology clinical practice guidelines and scientific statements within 2 years of publication, stratified by guideline, for guidelines published in 2016–2020.

Guideline*	Total Authors, No.	Paid Authors, No. (%)	Industry Payments Per Author, Median (Range), $
Lower Extremity Peripheral Artery Disease (2016)	21	7 (33)	4526 (1986–10,658)
Dual Antiplatelet Therapy in Patients With Coronary Artery Disease (Focused Update) (2016)	17	10 (59)	23,627 (724–65,302)
Adult Stroke Rehabilitation and Recovery (2016)	17	0 (0)	0 (0)
High Blood Pressure in Adults (2017)	21	3 (14)	99 (58–142)
Ventricular Arrhythmias and the Prevention of Sudden Cardiac Death (2017)	19	10 (53)	28,093 (2070–129,124)
Syncope (2017)	16	10 (63)	3926 (502–9233)
Clinical Practice Guideline Implementation Strategies (2017)	14	1 (7)	16,242 (n/a)
Use of Mechanical Circulatory Support: Ambulatory and Community Patient Care (2017)	14	8 (57)	6386 (689–15,162)
Cardiopulmonary Resuscitation in Adults and Children With Mechanical Circulatory Support (2017)	13	7 (54)	26,405 (10,967–39,965)
Blood Cholesterol (2018)	24	6 (25)	1500 (710–30,613)
Bradycardia and Cardiac Conduction Delay (2018)	19	7 (37)	1091 (235–4613)
Adults With Congenital Heart Disease (2018)	15	2 (13)	27,602 (15,640–39,565)
Innovations, Modifications, and Evolution of ACC/AHA Clinical Practice Guidelines (2019)	15	4 (27)	7329 (42–41,048)
Primary Prevention (2019)	18	2 (13)	16 (16–16)
Atrial Fibrillation (Focused Update) (2019)	15	8 (53)	1643 (121–32,451)
First aid: presyncope (2019)	9	0 (0)	0 (0)
Use of Advanced Airways, Vasopressors, and Extracorporeal Cardiopulmonary Resuscitation During Cardiac Arrest (2019)	11	2 (18)	709 (554–864)
Pediatric Advanced Life Support (2019)	11	0 (0)	0 (0)
Pediatric Basic Life Support (2019)	11	0 (0)	0 (0)
Cardiopulmonary Resuscitation and Emergency Cardiovascular Care (2019)	121	2 (2)	23,269 (22,480–24,059)
Early Management of Patients With Acute Ischemic Stroke (2019)	19	1 (5)	9705 (n/a)
Device Therapy and Arrhythmia Management in Left Ventricular Assist Device Recipients (2019)	21	5 (24)	46,982 (9796–107,207)
Cardiorenal Syndrome: Classification, Pathophysiology, Diagnosis, and Treatment Strategies (2019)	22	3 (14)	19,736 (9934–293,907)
Valvular Heart Disease (2020)	15	6 (40)	241 (178–813)
Hypertrophic Cardiomyopathy (2020)	19	8 (42)	940 (261–42,807)
Neonatal Resuscitation (2020)	10	0 (0)	0 (0)

*All guidelines and authorship information were retrieved from the American Heart Association (AHA) and the American College of Cardiology (ACC) websites. All payment data was extracted using the Open Payment Program database published by the Centers for Medicare & Medicaid Services.

## 4 Discussion

Our study of relationships with industry among cardiology guideline authors from 2014 to 2020 had several findings that advance current understanding of physician relationships with industry. First, we found that approximately 40% of authors per guideline received industry payments over the study period, and 25% received payments within 2 years of guideline publication. AHA and ACC requirements state that no more than 50% of authors per guideline can have a FCOI, and that authors with FCOI are prohibited from voting on recommendations that involve products developed by companies with which they have FCOI. Our findings suggest that these goals are being met. Second, we found that the average payment for a guideline author ($29,107) was around five times higher than previously reported average payments to all cardiologists (about $6500) [7]. There was also an unequal distribution of payments among authors, with over 80% of the total value of industry payments concentrated among less than 10% of authors. Since a small number of authors receive the vast majority of payments, the AHA and ACC could consider limiting involvement of high-paid authors based on thresholds of FCOI, in order to reduce potential or perceived bias by those authors. Third, we found no change in the number of guideline authors who received payments or the median sum of payments made to authors over the study period, suggesting that increased transparency of industry payments from the inception of OPP may not have impacted the relationship of guideline authors with industry. Finally, we found that 55% of authors who received industry payments within 2 years of guideline publication had FCOI disclosures. Prior studies investigating relationships between authors of guidelines in other specialties demonstrated FCOI disclosures only corroborate with OPP payment records up to one-third of the time [2,4]. The higher rate of concordance between OPP payments and FCOI disclosures among cardiology guideline authors may reflect stronger disclosure policies and enforcement by the AHA and ACC; however, a need for additional monitoring and compliance of FCOI disclosures remains.

This study has limitations. First, there may be inherent inaccuracies in payments reported by the OPP as well as limitations to OPP capture. As per OPP, payments made to any physician, physician assistant, nurse practitioner, clinical nurse specialist, certified registered nurse anesthetist, or certified nurse-midwife must be reported. Hence, any recipients of compensation who do not fall within these “covered recipients”, as defined by OPP, are not accounted for. Second, we used comprehensive (rather than guideline-specific) FCOI information provided by guideline authors, which may overestimate the concordance rate between FCOI disclosure and industry payments. Third, non-monetary compensation such as food and beverage may not have been viewed as relationships with industry by guideline authors but are documented as OPP general payments, which may overestimate the discrepancy between FCOI disclosures and industry payments.

## Author contribution statement

Subhanik Purkayastha: Conceived and designed the experiments; Performed the experiments; Analyzed and interpreted the data; Wrote the paper. Ruina Zhang: Conceived and designed the experiments; Analyzed and interpreted the data; Wrote the paper. Xiaohan Ying: Conceived and designed the experiments; Contributed reagents, materials, analysis tools or data. Vinay Kini: Conceived and designed the experiments; Analyzed and interpreted the data; Contributed reagents, materials, analysis tools or data; Wrote the paper.

## Data availability statement

Data will be made available on request.

## Additional information

No additional information is available for this paper.

## Declaration of competing interest

The authors declare that they have no known competing financial interests or personal relationships that could have appeared to influence the work reported in this paper.
